# Voices of youth: youth participation in the CO-CREATE project

**DOI:** 10.1186/s12889-025-23097-1

**Published:** 2025-05-20

**Authors:** Biljana Meshkovska, Gerben Moerman, Evelyne Baillergeau, Christian Bröer, Nanna Lien, Aleksandra Luszczynska, Ana Isabel Rito, Cécile Knai, Arnfinn Helleve

**Affiliations:** 1 Department of Nutrition, Institute of Basic Medical Sciences, Faculty of Medicine, University of Oslo, Blindern, P.O.Box 1046, Oslo, 0316 Norway; 2 Centre for Global Health, Sustainable Health Unit, University of Oslo, Oslo, Norway; 3 Department of Sociology, Faculty of Social and Behavioural Sciences, University of Amsterdam, Amsterdam, Netherlands; 4 Psychology Department in Wroclaw, SWPS University, Wroclaw, Poland; 5 Melbourne School of Psychological Sciences, Melbourne Centre for Behavior Change, University of Melbourne, Melbourne, Australia; 6 WHO Collaborating Center for Nutrition and Childhood Obesity - National Institute of Health Dr. Ricardo Jorge, Lisbon, Portugal; 7 Health Services Research and Policy, London School of Hygiene & Tropical Medicine, Faculty of Public Health and Policy, London, UK; 8 Centre for Evaluation of Public Health Measures, Norwegian Institute of Public Health, Oslo, Norway

**Keywords:** Youth, Participatory action research, Policy, Prevention, Overweight and obesity, Qualitative

## Abstract

**Introduction:**

Applying youth participatory action research (YPAR) has become common practice in public health. However, there are challenges in applying YPAR and there is a need for improving the process by learning from youth that has participated in such projects. This study explores youth involvement in the CO-CREATE project which focused on developing overweight and obesity prevention policies.

**Methods:**

This is a qualitative study, based on three data sources: 1) fieldnotes taken by project staff during observation of youth (approximately 150 observations) 2) semi-structured interviews with project staff (*n* = 12) 3) direct feedback from participating youth through feedback forms (*n* = 51). Data was analyzed in NVivo, following the principles of reflexive thematic analysis.

**Results:**

Five themes were generated which showcase youth involvement in CO-CREATE: 1) an inspired and engaged youth; 2) insights through respectful interactions; 3) a strength of voice and call to action; 4) challenges in understanding project and disengagement; and 5) interpersonal conflict and pressure to participate.

**Conclusion:**

Taking the time to build trust and create a feeling of equality is important. It is also important to allow youth to take the lead when they wish so and have clear ideas of how to do so. Voices of rebelliousness can be a show of strength and help reach the goals of the project. Participation in CO-CREATE may have contributed toward individual empowerment of youth.

**Supplementary Information:**

The online version contains supplementary material available at 10.1186/s12889-025-23097-1.

## Introduction

The United Nations Convention on the Rights of the Child [1] marked the starting point when children and adolescents stopped being merely the target of research and were viewed as able participants in discussions concerning their well-being [2]. Together with the movement toward participatory action research (PAR) [3] there has been a greater application of youth participatory action research (YPAR) specifically in areas such as public health [4]. YPAR entails the engagement and empowerment of children and adolescents working toward social change and community development, through four general phases: choosing a topic of focus, conducting research, analyzing findings and activism based on the findings [4]. However, YPAR is not without its challenges, and there are calls for more research on the different methods through which children and adolescents can be involved, need of information about the stages of the project in which they would participate, and finally, more knowledge on what the benefits of participation may be for youth [5].

To properly design and implement projects which apply YPAR, it is crucial to learn from the participation related experience of youth themselves, which is lacking [6]. One way to understand their experiences is through identifying and mapping their behaviors and emotions when taking part in projects. Emotions although somewhat neglected in the study of political actions and social movements throughout the 1970s, have become the focus of research again in recent decades [7–10], the drive being the realization that emotions simply pervade political (and social) action [7]. With this in mind, identifying and gaining a deeper understanding of the behaviors and emotions of youth throughout their activism and participation in researcher-initiated projects, would offer valuable insight on how YPAR principles could be improved.

Obesity has been identified as a key risk factor for non-communicable diseases (NCDs) [11]. In particular, overweight and obesity are an ongoing challenge for children and adolescents affecting one in three children in the WHO European region [11]. The CO-CREATE project (Confronting obesity: Co-creating policy with youth; www.co-create.eu; 2018–2023) was launched, with the overall aim of using policies that promote healthier food intake and physical activity environments to reduce overweight and obesity among adolescents in Europe [12]. Based on this overall aim, two main sub-aims were specified: (1) recruiting and working with youth to develop new policies which may address the issue of overweight and obesity and (2) through the research conducted as part of this project develop new methods for monitoring, benchmarking and evaluating existing policies relevant to the issue of overweight and obesity [12]. The project was conducted in five countries: the Netherlands, Norway, Poland, Portugal, and the United Kingdom [12]. To ensure youth involvement throughout the entire project duration, PRESS (the youth organization of Save the Children Norway) were partners and part of the project consortium, involved from the planning stages onward [12].

The work with youth was based on the principles of YPAR [4] and entailed forming youth alliances, where adolescents and adult researchers worked together toward the goals of the project which was generating, refining and finalizing policy ideas to address overweight and obesity [13]. A detailed policy form was developed and used by the alliances to capture policy ideas of the youth and help guide their development and implementation [13]. Different activities were implemented by the youth alliances at different stages of the project with (1) group model building and system mapping activities for the generating policy ideas phase, (2) conversational interviewing, photovoice, budgeting and trying ideas for the refining policy ideas phase and (3) finalizing the policy form, dialogue fora and reflection as part of the finalizing policy ideas phase [13].

The specific aim of this paper is to: map perceived and expressed behaviors and emotions of youth, and in this way explore the involvement of adolescents in a research project focused on developing overweight and obesity prevention policies. Namely, we look at both positive and negative experiences of youth throughout their involvement in youth groups (alliances) working to develop policies to prevent overweight and obesity as part of the CO-CREATE project. We conceptualize emotions as follows: ‘events elicit feeling, feeling organizes expressions (or outward signs) of feeling and automatic and instrumental behavioral reactions designed both to manifest outwardly and, in addition, to deal with the feeling’ [14].

A full and more detailed description of the CO-CREATE project as well as the youth alliances, including a detailed overview of the recruitment, demographic profile of participants and number of meetings held is published elsewhere [12, 13, 15].

## Methods

### Participants and procedures

There were approximately 15 youth alliances with 199 members, aged 15–19 [15], across the five countries of the project [13]. Approximately 10 meetings were held per alliance and meeting times varied across alliances and countries. Recruitment was done through municipalities, schools and local youth organizations (e.g. scout groups in Portugal), and efforts were made to ensure diversity in regard to social background, although this was challenging [13]. In the cases where recruitment was done through schools (this was the case with the Netherlands and Poland) youth participants may have already known each other. This was also the case in Portugal, where participants knew each other through the scout groups. In other situations, youth did not know each other prior to the project. When the alliances were held on school premises (the Netherlands, Norway and Poland) teachers may have been present in exceptional circumstances only, that teachers were absent was the default premise of the project design.

Each alliance also had a minimum of two adult project staff members identified as a facilitator (from the CO-CREATE consortium partners) and a co-facilitator (younger staff member) [13]. Although not all facilitators may have had experience working with youth, the purpose of the co-facilitators (youth) was to serve as a ‘bridge’ between adult project staff and participants [13]. Co-facilitators in most cases did not know the facilitators prior to the start of the project. However, co-facilitators participated in a two day training to become familiar with the project and get to know facilitators and other project staff [13].

Alliances were held in the local language, and implemented throughout the 2019–2020 school year [13]. Meetings of the alliances began in person, but due to COVID-19 were shifted online as the project progressed.

### Data for this study

Three different data sources were used in the current study, all produced in the context of or immediately following the completion of the work of the youth alliances. The first data source is the fieldnotes written up by facilitators based on observation as well as minutes taken by co-facilitators during youth alliance meetings. Although the number of meetings of each youth alliance varied across the five countries, approximately 30 meeting observations in the form of fieldnotes were gathered per country [13]. The fieldnotes, which were written in English, were structured around mainly pre-defined and generated topics (43 topics in total) which were reported elsewhere [13]. The pre-defined topics were identified after discussions among the project team as to what they thought were the most relevant areas to observe and report on during the alliances. These topics were operationalized on the basis of six research questions that examined how youth engagement strategies influenced the formation and sustainability of the alliances. The aim was to observe how interactions shaped young people’s political agency, problem perceptions and development of policy ideas. In this paper, we analyze data on three of the topics: (1) general observations on group dynamics during the alliances (2) challenges throughout the alliance sessions and (3) ethical issues that arose during alliance meetings. Facilitators were given extensive guidance and training on what to include under each topic. For example, in filling in the topic *group dynamics*, facilitators were asked to ‘describe the dynamics of the group’. In regard to *challenges* the main question was to ‘describe challenges explicitly raised during the meeting’. Finally linked to *ethical questions* facilitators were asked ‘did you observe that adolescents misunderstood their role as research participant or misunderstood the conditions for their participation in the alliance’. These are only examples of one of the main questions asked under each topic. Additional questions and follow ups were also asked.

The second data source consists of semi-structured interviews which were conducted with co-facilitators by research staff from the project, at the completion of the work of the youth alliances. Interviews were conducted in English. Interviews were not recorded, but notes were taken which are subject to analysis here. A total of 12 interviews were conducted (*n* = 2 from the Netherlands, *n* = 5 from Poland, *n* = 3 from Portugal, *n* = 2 from the UK). The following are some of the topics covered in the interviews: reflections on the role of co-facilitator, experience of taking minutes during the youth alliances, what worked and what didn’t work, relationships with participants, views on YPAR among participants and reflections on the overall experience.

Finally, the last data source comprises the feedback form developed for this project and filled out by the adolescents after the completion of the last meeting of the youth alliances. The forms were filled in in the native languages and translated by the co-authors with relevant language knowledge. Feedback forms were collected from youth alliance members in Norway (*n* = 5), Poland (*n* = 17), Portugal (*n* = 15) and the Netherlands (*n* = 14). The forms had open ended questions, giving the opportunity to gather qualitative data. Participants were asked about their overall experience participating in CO-CREATE, about particular activities they liked and didn’t like, about the topic of obesity and its relevance for their age group, about project staff, as well as their thoughts on whether the project work would continue after the official end of the youth alliances. The feedback form is available in full as Supplementary file 1.

Although the data of the fieldnotes has been used in varying degrees, to answer different research questions as part of other research [13, 15–17], the qualitative data from the interviews as well as the feedback forms have only been used for the purpose of the current paper.

### Analysis

The data from the fieldnotes linked to topics and subtopics of ‘group dynamics’, ‘challenges’ and ‘ethical questions’, as well as the notes from the interviews and qualitative answers as part of the feedback forms were all uploaded to NVivo for analysis. For the analysis we followed principles of reflexive thematic analysis [18]. Thematic analysis gives the possibility of finding and making sense of patterns of meaning in a data set, and consists of six phases: 1) familiarization with the data, 2) coding, 3) generating themes, 4) developing and reviewing themes, 5) refining, defining and naming themes and 6) finally write up [18]. We will elaborate on our steps in greater detail below. Firstly, BM read and re-read all the qualitative data from the three data sources. Secondly, to ensure that we work with data that exhibits the experience of youth most closely, after thorough discussion, we (BM, NL, GM, AH, CB, EB) decided to code all the qualitative data based on the perceived and expressed emotions and perceived and exhibited behaviors (such as being talkative, engaged) by the youth which were described by facilitators, co-facilitators and the youth themselves. Thirdly, the data was coded by BM. For example, data was coded where participants expressed or were described as being angry, confused, bored, fearful, or exhibited behaviors such as enthusiasm, engagement, resistance, collaboration, respectfulness. A total of 78 codes (behaviors and emotions) were compiled. Fourthly BM looked at patterns and commonalities of the situations where certain types of behaviors and emotions were perceived and/or exhibited and then mapped behaviors and emotions under the five themes presented in the results (please see mind maps created at this stage, as Supplementary files 2–6). The themes were discussed among the co-authors (BM, NL, GM, AH), refined and named and finally, the paper was written up by BM. Theme names were revised following suggestions of all co-authors, throughout the process of revising the paper. Each theme also reflects a loose link with level of commitment of the youth to the project. For example, one of the codes under the theme *an inspired youth* is ‘engaged’, which is one of the strongest behaviors portraying commitment to the project identified from the data. We found that a common pattern that led to this engagement by the youth was having been given specific tasks, which they found useful. Thus, in the theme *an inspired youth* we collected behaviors and emotions which showed the strongest level of (qualitatively) determined commitment of the youth to the project. Finally, we went through several reiterations of the names of the final themes, before finalizing them in the current version.

### Reflexivity

One of the basic tenants of reflexive thematic analysis is subjectivity. In this work, we do not view subjectivity of the first author (BM) conducting the coding and analysis as a challenge of the research, but rather as something valuable. Subjectivity in this context is viewed as a unique perspective and the reflections and conclusions drawn are used to enrich the analysis and offer an in-depth account of the data [18]. BM is a qualitative researcher in her 40s with a background in policy analysis and implementation science. Although familiar with the CO-CREATE project through its duration, she was not directly involved in any of the official project activities. As such, she positioned herself as somewhat of an outsider when beginning this research, who would look at the data with a fresh eye, identify the places where voices of the youth were audible, and try to interpret based on the identified patterns. She does know some of the other co-authors through previous collaborations (from Norway, Poland), while the rest she met through the work on this specific research (from the Netherlands, Portugal, UK). At the same time, the remaining co-authors are senior researchers, professors, academics and practitioners, qualitative and quantitative researchers from a variety of fields, all directly involved in CO-CREATE for the duration of the project. Age range of the co-authors is 40–60. They are familiar with the activities as well as the data, as some of them were also facilitators in the project. As such, they were able to reflect on the findings on an ongoing basis and give their feedback and input to ensure that the way that the data was being interpreted was not at fundamental odds with their own experiences. With this combination of the outsider / insider perspective to the data, we hope that we capture the voices of youth as much as possible, and do justice to how the youth experienced their participation in the project.

### Ethics

At the start of the project the partners developed an ethics policy which consisted of six considerations: (1) voluntary participation, (2) protection from obesity stigma, (3) respect for young people’s time, (4) data privacy and confidentiality, (5) power balance and (6) equal opportunity to participate in the research [16]. These are discussed in full elsewhere [16].

## Results

Five overarching themes were generated from the analysis that explore youth involvement in CO-CREATE. The following themes are discussed: 1) an inspired and engaged youth; 2) insights through respectful interactions; 3) a strength of voice and call to action; 4) challenges in understanding project and disengagement and finally, 5) interpersonal conflict and pressure to participate. A description of each theme is presented in Table 1.

**Table 1:** Themes with short description

Theme	Description
An inspired and engaged youth	This theme reflected positive emotions and behaviors as well as high commitment to project work by the youth.
Insights through respectful interactions	Under this theme, youth showed more subdued enthusiasm. The highlight was an emphasis on personal lessons learned for the youth.
A strength of voice and call to action	The theme reflected the voice of youth very clearly. Under this theme youth showed their strength and creativity through rebelliousness and calls to action.
Challenges in understanding project and disengagement	This theme was dominated by low engagement in the project, feelings of indifference, shyness and at times confusion about the aim and methods of the project by youth.
Interpersonal conflict and pressure to participate	The final theme reflected on situations of conflict among youth participants and between youth and teachers (in the exceptional situations when teachers were involved)

### An inspired and engaged youth

The theme *an inspired and engaged youth* is dominated by positive behaviors and emotions by the youth, in situations that showed high commitment to the project work. Some examples are the following: being active in between project meetings, independence, leadership, fighting for an idea, care about project, feeling like equal partners, trust, being supportive of each other, sharing personal experiences, talkative, engaged, enthusiastic and having fun.

We found that participants were engaged when they were working on specific tasks, with clear guidance on how to conduct those tasks as well as what the purpose of doing those tasks was. In this regard, we recognize that engagement was a two way exchange, and the result of efforts by both the participants and the facilitators. For example, participants from across all countries were engaged when working on designing their own policy idea, constructing a systems map, using photovoice and learning to fill in a policy form, to name just a few examples (feedback form Norway, fieldnotes Norway, fieldnotes UK, fieldnotes Portugal, fieldnotes Poland, fieldnotes Netherlands). There was no overall agreement on the usefulness of specific tasks, for example photovoice was strongly liked by some and disliked by others. The lesson learned was that particular organized activities as part of the project that were new but found to be useful to the participants, were appreciated. Participants expressed enthusiasm when they learned new skills as well, such as how to present their own ideas, speaking to politicians, writing letters to stakeholders (fieldnotes Norway, feedback forms Netherlands, feedback form Poland). In addition to engagement and enthusiasm, situations where participants were learning a new task or skill were also described as fun (feedback form Norway). However, the knowledge in conducting new tasks and the skills learned were most appreciated when participants could make a clear link between what they were learning, the overall aim of the project, and the usefulness of it all in their own life and for their own future.

In situations where there was high commitment to the project, the relationships between participants, as well as between participants and project staff were based on trust, support and participants expressed feeling they were on an equal footing with project staff. Overall, the opportunity to do group work was one of the most appreciated aspects of the project as expressed by youth:

*“Only positive things*,* I’m glad that I had the opportunity to meet so many wonderful people and I hope that this is just the beginning” (feedback form Poland)*.

*“Very nice because one gets to be social and make new friends and learn some about political methods and be part of a group where nobody is the boss*,* but all equal” (feedback form Norway)*.

The expressed feeling of being equal with project staff, that ‘they are with us and not just looking after us’ (feedback form Norway) took time to establish through building trust and was partially due to participants seeing the project as not linked with school. Based on fieldnotes from all participating countries, trust was built through bonding moments both during but also outside of the context of the project meetings, when staff met with participants and created more informal opportunities for interaction and small talk. It was in the more informal context that participants for example proposed using a group application as a way to communicate, rather than the school email where they were not as responsive (fieldnotes Netherlands). That time was needed to build trust and create the feeling of equality, was further explained by project staff. Project staff stated that at the beginning of the project there were many rules to be set, which may have resulted in a misbalanced relationship between staff and participants (fieldnotes Netherlands). However, as the more active parts of the project started, which required action by the participants, the relationship dynamic changed, and a more equal bond was established as commitment to the project grew:

*“We all grew in terms of knowledge*,* wisdom but mostly*,* on how to give voice to a cause that initially might not be so close to us but that at the end has become something that we want to become a reality” (feedback form Portugal)*.

Trust was felt not only between participants and project staff but also among the participants themselves. For example, in one youth group participants expressed that they felt safe enough to share personal experiences with overweight, mental health challenges and bullying (fieldnotes Norway). A close relationship among peers also meant that they were supportive of each other, building on each other’s contributions, thus making the overall project work productive and smooth (fieldnotes from all countries, co-facilitator interview Portugal).

Finally, coming into the project with prior understanding of the topic of obesity and overweight, and an interest to work on this topic was common to some participants who emerged as leaders and worked well independently. They expressed care for the project and were willing to fight for their policy ideas and carry them forward. In these cases, participants who emerged as leaders made significant contributions toward the project goals with the potential of continuing the work beyond the end of CO-CREATE. For example, one particularly active participant with some prior knowledge of the topic, completed a task (filling in a policy form) independently and outside the timeframe of the regular sessions of the project, thus indicating a strong commitment (fieldnotes Poland). Project staff also found that those who showed interest in the project topic from the very beginning had a greater tendency to work in between sessions (fieldnotes Poland). However, activity by the participants in between sessions was also nurtured through efforts of project staff who regularly sent messages through social media (fieldnotes Poland).

### Insights through respectful interactions

Throughout the project, there were also emotions and behaviors that were more subdued. The underlying patterns in these situations reflected potential benefit for participants linked to their *insights gained*,* through respectful interactions*. Examples of such behaviors and emotions were: respectfulness, politeness, kindness, feeling safe, calm, comfort, deeper understanding of the project topic and raising awareness. Although this may not necessarily translate into significant contribution toward working on the project long term, it is important at the personal level.

As noted, project staff and participants across countries, on numerous occasions, described a working atmosphere that felt safe and comfortable, where interactions were based on respect, being polite and kind, and mutual collaboration on the work being done. In these situations, the benefit for participants was a greater understanding of the project topic, how it relates to their age group and their own lives (all countries with feedback form):

*“I would recommend taking part*,* because every young person should be aware of the problem of obesity among adolescents and have at least the basic information about it. In addition*,* it is the opportunity to do something good with great people in a relaxed atmosphere.” (feedback form Poland)*.

*“I began to notice many aspects of a social life that I hadn’t noticed before*,* e.g. designing public space that promotes unhealthy eating habits and*,* as a consequence*,* obesity.” (feedback form Poland)*.

As described across countries, one of the messages of the project was a systemic understanding of the issue of overweight and obesity rather than seeing it as an individual problem and responsibility. For some of the participants, this was a clear lesson learned:

*“Before integrating the CO-CREATE project*,* I considered that our lifestyle habits (eating habits*,* physical activity) were only dependent on each one of us. However*,* after the debates*,* I realized that our attitudes and values have an effect on these habits*,* although they are not the only factor. Society as a whole (financial situation*,* advertising*,* supermarkets*,* green spaces) influence our habits in a certain way. In this way*,* I realized that obesity is much more complex than I thought and that solutions have several paths” (feedback form Portugal)*.

For others, what they learned had an influence on their own behavior and habits:

*“I think that I changed a little bit*,* above all I have become more conscious how many people struggle with overweight and lack of exercise. My way of thinking has changed now*,* I think more about it*,* I need to practice more and not give up when I don’t feel like exercising.” (feedback form Poland)*.

*“Through this project*,* I have done a little more research on healthy lifestyle and healthy eating*,* and am more into this: ” (feedback form Netherlands)*.

Finally, awareness was raised that youth can also have a voice in the political arena, and that change is possible through activism. Even though this awareness may not come with a clear commitment to action, it is again, significant for participant’s personal gain (feedback form Netherlands, feedback form, Poland).

### A strength of voice and call to action

What also came to the surface throughout the project was the *strength of voice and call to action* of youth characterized by a rebelliousness with constructive undertones. This was expressed through emotions and behaviors such as resistance and even anger, doubt, and demanding action rather than talk from the adults as well as asking challenging questions.

Participants did not shy away from voicing doubts about particular aspects of the project. For example, some participants questioned the relevance of skills being taught (such as interviewing) and specific tasks such as the group assignments as part of the project, even as far as questioning the relevance of certificates of participation that were offered by the project (fieldnotes Netherlands). There were discussions regarding the topic of obesity and overweight and the degree to which this was a systemic issue. In many of these occasions, participants came to a new understanding of the topic and were accepting of the explanations offered by project staff.

There were also situations where youth made it clear when something was not acceptable. Participants had clear and strong preferences when it came to their pictures (not) being taken (fieldnotes Netherlands, fieldnotes Poland, interview co-facilitator Poland). Several forms of resistance were noticed toward project staff presence. On some occasions project staff observing the work of groups of participants was perceived as controlling, and staff was told that they were interfering with the work of the youth (fieldnotes Netherlands).

Long explanations and repetitiveness by project staff were not appreciated, rather a focus on what was perceived as useful content for the project work was called for (fieldnotes Norway, fieldnotes Netherlands). In some instances, project staff advice was understood by the youth as being ‘instructed’ on what they had to do, which was not in line with their understanding of the spirit of the project (fieldnotes Netherlands, fieldnotes Portugal). The objections were either voiced clearly or manifested by loss of attention and interest by the youth in these situations (fieldnotes Norway, fieldnotes Netherlands). When project staff gave policy related advice which clashed with the ideas of the participants, this was resisted (fieldnotes Norway). On some occasions, when staff gave the advice to take it slow and prepare before implementing any project related actions, this was not followed, and participants proceeded with their own (shorter) timelines (fieldnotes Poland, interview with co-facilitator Poland, fieldnotes Netherlands).

Finally, linked to this last point is the preference for action, demanding steps be implemented sooner rather than later (interview co-facilitator Portugal, fieldnotes Netherlands, fieldnotes Poland, interview co-facilitator Poland). One particular example portrays this preference clearly. A group of participants wanted to implement a policy idea which entailed cooking healthy food in a school canteen (fieldnotes Netherlands). They encountered resistance from the teachers that were involved in the management of the canteen, as well as from project staff who advised to take it slow and prepare before moving forward. Teachers were particularly doubtful, not only in regard to the implementation of the idea, but about the promised clean up as well. Participants however were persistent, and successfully delivered in all aspects. Once the activity was concluded, participants expressed feeling vindicated, and voiced ‘you had underestimated us’ several times (fieldnotes Netherlands).

### Challenges in understanding project and disengagement

Under the theme *challenges in understanding project and disengagement*, the predominant behaviors and emotions were of indifference, disengagement, confusion, feeling overwhelmed, struggling, distrust, shyness, fear of criticism, fear of speaking out and lack of commitment.

According to some participants, the topic of overweight and obesity did not feel relevant to their age group (feedback forms Portugal). In a similar direction, doubts about the systemic origins of the problem of overweight and obesity remained:

*“In this age group I find it difficult to be tackled*,* if not with activities of this kind. To tackle this problem*,* I think the parents are the ones who have to be more positively aware of how to feed their children. If they are used to healthy eating from an early age*,* they will not face these problems in this age group.” (feedback form Portugal)*.

For some of the participants, the topic was simply not interesting enough (fieldnotes Netherlands, fieldnotes Poland). Although for part of the youth certain project activities were seen as especially engaging those same activities were perceived as either difficult to understand, or simply not relevant by others (feedback form Poland, fieldnotes Netherlands, fieldnotes Poland, fieldnotes UK):

*“Photovoice*,* I will not hide that it was a bit pointless and boring for me.” (feedback form Poland)*.

Challenges with understanding basic concepts within the project, such as what a policy idea is, were also reported (fieldnotes Netherlands, fieldnotes Norway, fieldnotes Poland, fieldnotes UK). In many situations, insecurities with their level of knowledge and understanding may have led to participants expressing fear of speaking out and shyness (fieldnotes UK, fieldnotes Netherlands, fieldnotes Poland). In such situations project work could only proceed with significant encouragement and support from project staff. Another pattern at the heart of these behaviors and emotions was that participants could not see the long-term goals of the project, and could not link the work of the project and its relevance to their own life and goals (interview with co-facilitators Netherlands, fieldnotes Netherlands). In this direction, it was not helpful that an overall overview of the project timeline was sometimes lacking:

*“I think we could have known the topic of the next meeting at the previous meeting so that we could be able to prepare something at home and not “waste” so much time at the meeting.” (interview with co-facilitators Netherlands)*.

Another challenging pattern underlying this theme was the perception of project staff as teachers (fieldnotes Poland, fieldnotes Netherlands, fieldnotes UK). Project staff sometimes found themselves in the role of checking the work of participants, which was perceived as controlling (fieldnotes Poland, fieldnotes Netherlands, interview with co-facilitators UK). In some specific cases, project staff reported that simply due to their level of knowledge and experience, they may have influenced the work of participants and shaped their policy ideas (fieldnotes Portugal, co-facilitator interview UK):

*“A phd. have bias*,* have different understanding (…) come from different world*,* academic” (co-facilitator interviews UK)*.

Struggles with regards to who holds authority and how authority is shared, if at all, were particularly pronounced where school teachers were involved. This was especially so when teachers intervened or made decisions without consulting participants or project staff (fieldnotes Netherlands, fieldnotes Poland). However, this was in exceptional and few circumstances. In one particular example, teachers decided to remove participants from the project without consulting project staff (fieldnotes Netherlands). There was a feeling of distrust when teachers taking part in the project transferred the school culture to the project, in particular when the two did not match (fieldnotes Netherlands). It should be noted that in the latter case, youth alliances were implemented in the schools and during school hours, which is likely to have the transfer of school culture to the project.

Finally, school and school obligations had a lasting presence throughout the project, as participants would miss project meetings or would explain lack of engagement due to schoolwork (feedback forms Portugal, feedback forms Netherlands, fieldnotes Norway, fieldnotes Poland, fieldnotes Netherlands, fieldnotes UK, interview with co-facilitator Poland). Schoolwork not only influenced the engagement of participants throughout the project, but also called into question their commitment to continuing the work on their policy ideas after the end of the official timeframe of the project:

*“I would like to continue but I say no*,* because this year will be an important school year for me and I already notice that I spend a lot of time on school. If I were to continue*,* I really want to invest my time in it*,* but because I have to work*,* play sports and train alongside school*,* I know that this won’t work out. During the week I’m busy with school every day and in the weekend I only have one day off*,* in which I often have to work on school as well.” (feedback form Netherlands)*.

It should be emphasized however, that minimal interference in and prioritization of schoolwork over the project was envisioned from the start of the project. Working on CO-CREATE was never meant to ask participants that they neglect their studies in any way.

### Interpersonal conflict and pressure to participate

Finally, the last theme is characterized by negative behaviors and emotions such as dissatisfaction, disrespect, bullying, feeling annoyed, arguing, disturbing and feeling forced to participate.

There were several types of challenges in regard to interactions between participants. In some cases, participants had clear preferences of who they wanted to work together with, and were dissatisfied when this was not possible (fieldnotes UK, fieldnotes Netherlands). In other situations, when participants knew each other and were in conflict prior to the project, this spilled into the project work. For some, working with peers was simply not of interest:

*“I didn’t particularly like working together*,* still don’t*,* but I do accept now that you have to do something with everyone” (feedback form Netherlands)*.

In the same direction, a few instances of bullying language and behavior were also noticed (fieldnotes Portugal, fieldnotes Netherlands). For example, one participant laughed at another when the latter was looking at and trying to understand a system map (fieldnotes Netherlands). Participants also showed annoyance when project staff would pay too much attention to some participants simply because they were obstructive, rather than to all participants equally (fieldnotes Netherlands). Few instances were noted where participants were disturbing the work of the others (fieldnotes Netherlands).

When some participants were less active, this could pull others into the same behavior (fieldnotes Netherlands, fieldnotes UK). In fact, one of the main criticisms the project received was that participants did not appreciate when some of their peers were less active, missed sessions or simply did not do the work (feedback form Poland, feedback form Portugal):

*“Maybe I would throw out the people who don’t do anything.” (feedback form Poland)*.

*“At the end I was not so motivated*,* also due to Covid-19*,* so I started to get a bit detached. I also felt that in the group there were only two or three colleagues interested in the project and this reduced even more my motivation.” (feedback form Portugal)*.

Finally, there was one instance in which a participant had expressed to staff that he was forced to participate, and participation was not voluntary (fieldnotes Netherlands). When project staff discussed the situation with teachers, they were informed that without project staff knowledge or approval, the project was linked to school activities which were mandatory. This was later rectified, and participants were informed that they did not have to participate in the project if they did not want to. However, this rather singular and extreme example, brings up the issue of consent of youth more generally. As part of this project, all participants were informed about the project, what their participation would entail, and signed consent forms. However, several challenges remained even when this process was strictly followed. Firstly, when project meetings are held on school grounds, which in many cases is simply safest and easiest for the youth themselves, it is not clear how much youth may be associating participation in the project with their regular schoolwork and consequently to what extent they may feel pressured into participating without realizing it (fieldnotes Poland). Even though in most cases project staff did their best to fully explain participation in the project, there were reports where this process for various reasons was rushed, and it remains unclear if youth understood what signing the consent forms meant (fieldnotes Norway). Emphasis seems to be on signing forms, and once forms are signed, the issue of consent is closed (fieldnotes Poland). The latter is particularly problematic considering, as raised by project staff, that participation in this project also meant youth were research subjects. Although they had been informed of this, question remains if they understood what that meant, and equally importantly, if they remembered throughout the duration of the project that they were also research subjects (fieldnotes Netherlands, fieldnotes Norway).

## Discussion

In this paper we map perceived and expressed behaviors and emotions of youth, and in this way explore the involvement of adolescents in a research project focused on developing overweight and obesity prevention policies. We found that when youth believes in and has a good understanding of the purpose of a project their commitment and work is more thorough. At the same time, we also identified situations of rebelliousness which was harnessed into action. Finally, there were occasions where youth did not feel a link to the project and felt it like a school related obligation rather than voluntary action. We explore some of these findings next as they relate to relevant existing literature.

### Trust and relationships matter

Overall, emotions and how they may shape the discourse and actions around a particular issue are a neglected aspect of social movements [7–9]. When part of a group, joy, hope, enthusiasm, pride, attachment to the group are seen as necessary emotions for the group to continue to exist [7, 9]. Research which explores expressed emotions throughout a YPAR process with youth in El Salvador finds that when the process is positive, trust toward others increases [19]. Similarly, other work found that when interactions are based on close personal ties that developed among participants lines between participants and facilitators were blurred, as facilitators were expected to take equal part in this process that required a ‘personal investment’ [10]. This helped in strengthening the group, and not losing participants throughout the entire duration of a project, which is often a challenge [10]. As our study found, more equal sharing of power between young participants and project staff was important. The opportunity to socialize with other young people was an important finding.

### The energizing power of rebellion

The literature also discusses the relevance of seemingly negative emotions, such as rebellious anger and how these may be nurtured into a strong commitment to a cause [20]. More generally, negative emotions are seen as powerful and necessary for mobilization to occur [9]. Anger, outrage, indignation, pride, may give rise to action [7]. This rebelliousness was visible in our work and of value as it pushed youth toward action that may have contributed toward their commitment to the project. Transforming shame into dignity and pride, are also identified as strong drivers of social movements [7]. In our research, this was an evident process through the example of youth moving forward with their plans and after being successful voicing the feeling of being vindicated. Other research warns against welcoming ‘convenient voices’ only, and not involving or not knowing how to involve ‘inconvenient voices’ [21]. Further, moments of disturbances and frictions, are valued as much as moments of joy, as these ‘productive tensions’ are what gives rise to new meanings and knowledge [22].

Consistent with our research, others have found that youth have a preference for methods that involve action and doing [19]. This is the case not only when working with youth, but also with adults with a low socioeconomic position, where when implementing community based programs, it is recommended that interested participants should start immediately, and not be made to wait [23]. As we found, speed, movement, action and not wasting too much time on theoretical thinking and planning is preferred. Too much of the latter, and there is the danger of loosing young participants along the way. That out of 199 adolescent participants in CO-CREATE at the very beginning of the project, only 51 filled in the feedback form, may indicate that some were lost along the way.

### When not being involved from the start

Shame, the feeling of inferiority, fear, are all present when there are power imbalances [20]. These types of emotions were also explored through our research and particularly visible where clear links between CO-CREATE and the school context and school staff existed. Emotions such as indifference, doubt and boredom were also identified. In CO-CREATE, youth was heavily involved in the project through the design of policy ideas, and efforts were made that there be power sharing and an equal partnership between youth and project staff. However, they were not involved in the very inception and design of the project from the start, in choosing the topic of focus and the methods used throughout the project. They were also not involved in the analysis of the research conducted as part of the project. This may have led to lack of commitment and low participation.

Concerning the topic of overweight and specifically obesity, youth expressed doubts about the systemic origins of obesity. Some persisted in attributing it to individual responsibility. Relevant research highlights the importance of ‘moral shocks’ in recruitment of participants in a social movement, that occurs when an unexpected event gives rise to a feeling of outrage, which brings about participation in a political action and thus becoming part of a social movement [7, 9, 24, 25]. What is important in this context is to also have someone to blame [7, 9]. If obesity is viewed and understood as individual responsibility, this aspect of having factors external to the individual to ‘blame’ was lacking from CO-CREATE for some of the participants, and may partially explain their lack of commitment. In the same direction, young people are not necessarily enthusiastic every time there is a chance to participate, as they may not feel comfortable, or they do not value the type of participation offered [21].

### Consent – revisit again and again and again

O’Farrelly and Tatlow-Golden discuss the issue of consent with children and adolescents. They distinguish between understanding what participation in a research project means, which is possibly higher among older children and adolescent groups, with the ability to practice agency in school contexts, which is arguably lower [26]. In this direction, a paper published based on the work done as part of the CO-CREATE project, reflecting in greater detail on the issue of consent, recommends that the age at which adolescents can give legal consent to participate in a project should be lowered from the usual 15–16 to 12 [27]. This would ensure that those that want to participate can do so, in situations where their parents or guardians may have declined.

In addition, although recommendations are that it is important to always think about ethical implications, throughout a project, and when it ends [22] research also recognizes that projects also deal with practical constraints and compromises must be made [10]. This fits in well with the consent related issues encountered throughout CO-CREATE. Although significant efforts were made to explain the research aspects of CO-CREATE to youth, as well as to make sure that they do not feel obligated to participate where recruitment was done through schools, this was not always successful. Namely, even when recruitment was not done through schools, project staff wondered if participants were fully aware that they were also being researched in the project, throughout its duration. This questions the practice that consent should be discussed only once, and whether such emphasis should be placed on signing forms rather than on gaining a deeper understanding of consent, and re-visiting the issue multiple times for the duration of a project.

### On empowerment

YPAR is specifically used with the aim of contributing toward empowerment of youth [28]. Empowerment is often conceptualized at three levels: individual, organizational and community [29]. Individual empowerment which is defined as the ability to make decisions and practice control over one’s life [29] is similar in meaning to constructs such as self-efficacy and self-esteem, developing a positive self-concept and personal competence [29]. It is this individual level of empowerment that has been mainly identified through this research and as a result of participation in the CO-CREATE project. This was evident throughout our findings and reports from youth that participation increased their knowledge on the topic of obesity. In addition, youth learned some new skills which they may continue using in their personal life. Youth reported stronger confidence when approaching policy makers and other stakeholders. Stronger leaders from the alliances emerged over time. That youth did not want too much help from the adults was also a sign of individual empowerment, and consistent with findings of other research linked to YPAR [30]. However, what was also consistent with other work was that when individuals who hold formal and informal power in certain contexts (such as teachers in the school) were involved in any way, conflict can arise [31]. This was particularly the case in the very few situations where teachers were involved in the recruitment linked to CO-CREATE and may have been present at some alliance meetings. As already stated, this was only in exceptional circumstances in the context of CO-CREATE as by design, teachers were not meant to participate or interfere in the work of youth.

### Strengths and limitations

This research aimed to capture the voices of youth, as both participants and target group of the project. CO-CREATE gave an opportunity to youth to participate in a policy related process and learn skills and gain knowledge which could be of use to their own futures, although that they were not involved in the conceptualization of the project may be a limitation. Although the data does reflect the views of youth, some of the data sources come from project staff, and thus, views of youth are interpreted through the perceptions of project staff. We tried to mitigate this as much as possible by focusing and isolating instance of behaviors and emotions of youth, in all the described situations. This was done specifically through the method of analysis used. Additionally, the feedback forms which were filled in by youth, were done by those that completed the entire process and took part in the project until the very end. Due to time and capacity constraints as well as the onset of the COVID-19 pandemic, no feedback forms were provided from the UK alliance. The voices of youth that dropped out are not represented through this data source.

## Conclusion

This research explored youth experiences when participating in the CO-CREATE project. Power sharing, equality and taking the time to build trust between participants and project staff are often highlighted as important. However, youth was most engaged when taking the lead in proposing and designing their own policy ideas as well as when learning new skills and gaining new knowledge which they found relevant to their own life and future. Voices of rebelliousness were identified here as a strength, and it is shown how through the drive toward action, they are critical in achieving the goals of the project.

## Electronic supplementary material

Below is the link to the electronic supplementary material.

Supplementary Material 1

Supplementary Material 2

Supplementary Material 3

Supplementary Material 4

Supplementary Material 5

Supplementary Material 6

## Data Availability

Raw data is not publicly available due to ethical and legal restrictions. The documentation of the analysis that support the findings of this study may be available from the corresponding author (B.M.) upon reasonable request. Analytic code availability: Analytic code used to conduct the analyses presented in this study are not available in a public archive. They may be available by emailing the corresponding author.

## Abbreviations

PAR: Participatory action research
YPAR: Youth participator action research
CO-CREATE: Confronting obesity: co-creating policy with youth
