# Supplementary material for: Voices of youth: youth participation in the CO-CREATE project
Source: BMC Public Health. 2025 May 20;25:1858. doi: 10.1186/s12889-025-23097-1 (PMC12090452; doi:10.1186/s12889-025-23097-1)
Supplement: Supplementary file 1 — Supplementary Material 1 [file 12889_2025_23097_MOESM1_ESM.docx]

| 2019 | 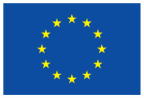This project has received funding from the European Union’s Horizon 2020 research and innovation programme under grant agreement No 774210 |
| --- | --- |

| **FEEDBACK FORM FOR CO-CREATE FOR ALLIANCE’S ACTIVITIES** |
| --- |

| Insert name of alliance and location here  30.04.2019 |
| --- |

­

Name (optional): _______________________________________________________________________

1. What is your overall experience participating in CoCreate activities?

|  |
| --- |

1. What are the activities you find the most valuable and why?

|  |
| --- |

1. What are the activities you find the least valuable and why?

|  |
| --- |

1. What are the changes you experienced throughout being involved in the activities?

|  |
| --- |

1. What are your views about the challenge of adolescents’ obesity?

|  |
| --- |

1. What do you think are adolescent’s roles in addressing adolescent’s obesity?

|  |
| --- |

1. What do you think of the facilitation of the meetings?

|  |
| --- |

1. What are your suggestions in making things better?

|  |
| --- |

1. Would you recommend these activities to your friends and why?

|  |
| --- |

1. Do you see your alliance to continue working together and how?

|  |
| --- |

Thank you very much for your feedback.
