# Supplementary figures and images for: Voices of youth: youth participation in the CO-CREATE project
Source: BMC Public Health. 2025 May 20;25:1858. doi: 10.1186/s12889-025-23097-1 (PMC12090452; doi:10.1186/s12889-025-23097-1)

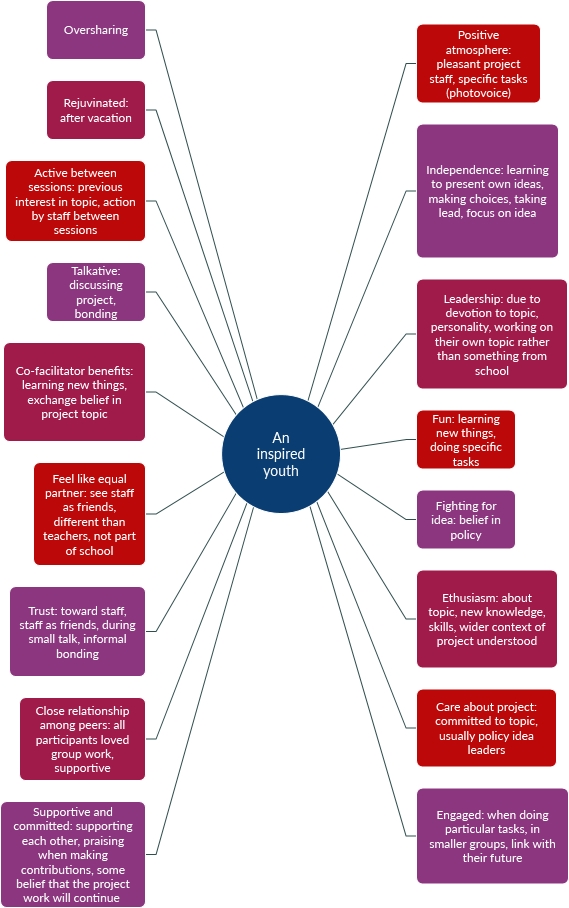

Supplement: Supplementary file 2 — Supplementary Material 2 [file 12889_2025_23097_MOESM2_ESM.jpg]

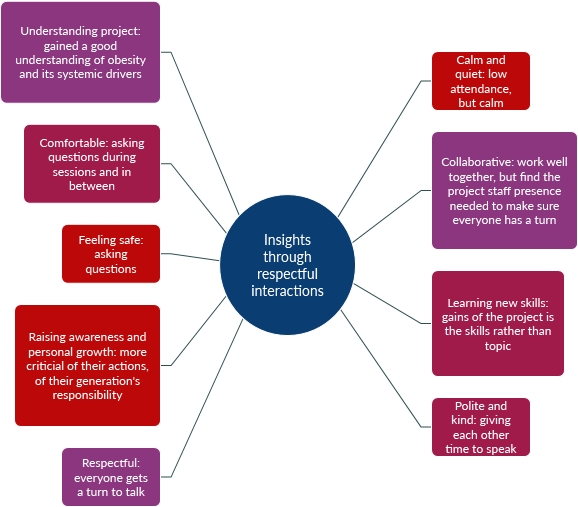

Supplement: Supplementary file 3 — Supplementary Material 3 [file 12889_2025_23097_MOESM3_ESM.jpg]

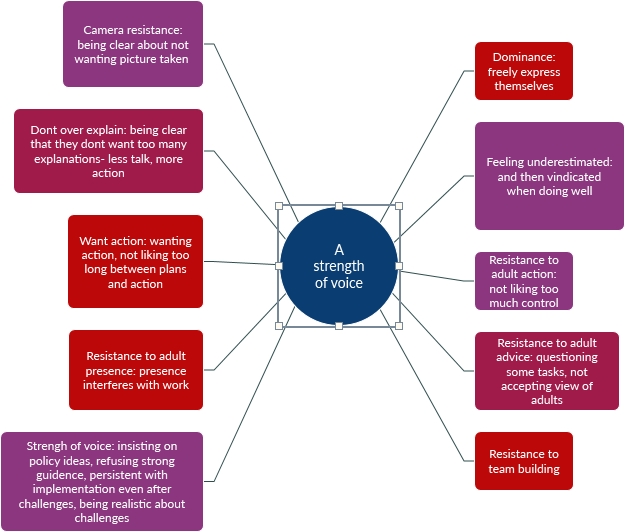

Supplement: Supplementary file 4 — Supplementary Material 4 [file 12889_2025_23097_MOESM4_ESM.jpg]

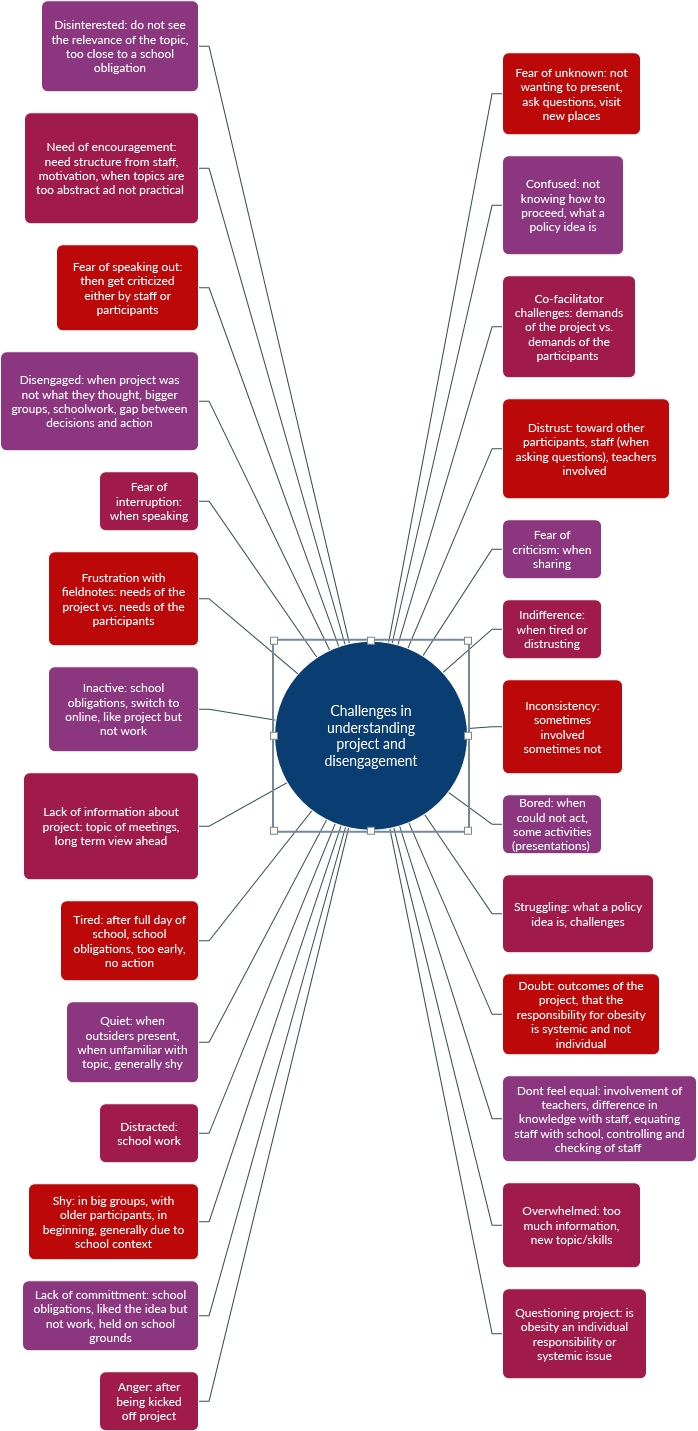

Supplement: Supplementary file 5 — Supplementary Material 5 [file 12889_2025_23097_MOESM5_ESM.jpg]

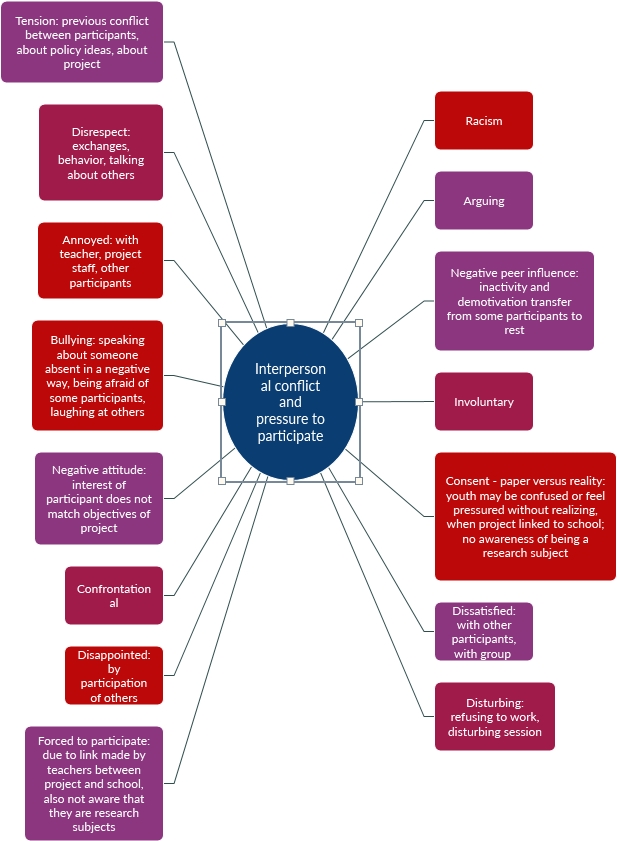

Supplement: Supplementary file 6 — Supplementary Material 6 [file 12889_2025_23097_MOESM6_ESM.jpg]
